# Understanding the Photoinduced Desorption and Oxidation of CO on Ru(0001) Using a Neural Network Potential Energy Surface

**DOI:** 10.1021/jacsau.4c00197

**Published:** 2024-05-10

**Authors:** Ivan Žugec, Auguste Tetenoire, Alberto S. Muzas, Yaolong Zhang, Bin Jiang, Maite Alducin, J. Iñaki Juaristi

**Affiliations:** † Centro de Física de Materiales CFM/MPC (CSIC-UPV/EHU), Paseo Manuel de Lardizabal 5, 20018 Donostia-San Sebastián, Spain; ‡ Donostia International Physics Center (DIPC), Paseo Manuel de Lardizabal 4, 20018 Donostia-San Sebastián, Spain; § Departamento de Polímeros y Materiales Avanzados: Física, Química y Tecnología, Facultad de Química, UPV/EHU, Apartado 1072, 20018 Donostia-San Sebastián, Spain; ∥ Key Laboratory of Precision and Intelligent Chemistry Department of Chemical Physics, University of Science and Technology of China, Hefei, Anhui 230026, China

**Keywords:** neural networks, femtochemistry, CO oxidation and desorption, Ru(0001), potential energy surface, laser-induced dynamics

## Abstract

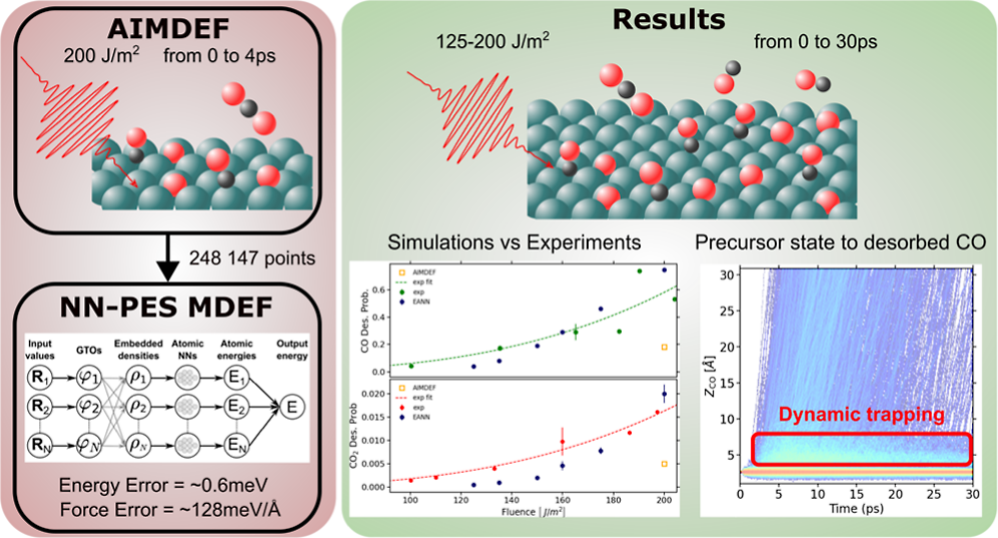

The study of ultrafast photoinduced dynamics of adsorbates on metal surfaces requires thorough investigation of laser-excited electrons and, in many cases, the highly excited surface lattice. While ab initio molecular dynamics with electronic friction and thermostats (*T*_e_, *T*_l_)-AIMDEF addresses such complex modeling, it imposes severe computational costs, hindering quantitative comparison with experimental desorption probabilities. In order to bypass this limitation, we utilize the embedded atom neural network method to construct a potential energy surface (PES) for the coadsorption of CO and O on Ru(0001). Our results demonstrate that this PES not only reproduces the short-time ab initio dynamics but is also able to yield statistically significant data for long lasting trajectories that correlate well with experimental findings. Furthermore, the analysis of the laser-induced dynamics reveals the existence of a dynamic trapping state that acts as a precursor for CO desorption, and it is not observed under thermal conditions. Altogether, our results validate the underlying theoretical framework, providing robust support for the description of not only the photoinduced desorption but also the oxidation of CO in terms of nonequilibrated but thermal hot electrons and phonons.

## 1 Introduction

It has been demonstrated that utilizing intense (∼100 J/m^2^) femtosecond laser pulses across ultraviolet, visible, and near-infrared wavelengths provides an efficient means of stimulating surface reactions in the presence of adsorbates on metal surfaces.^1−4^ Within this range of wavelengths, a significant fraction of light is absorbed by the metal, leading to electronic excitations. Following this, energy transfer to lattice atoms occurs via electron–phonon coupling, producing a combined electronic and phononic excited system that interacts with adsorbates. As a consequence, adsorbates may undergo diffusion, exhibit reactivity, or experience desorption. Notably, illuminating such systems with laser pulses can reveal new reaction pathways compared to conventional thermal excitation conditions. A paradigmatic example of these kinds of processes is the oxidation of CO on Ru(0001) under ultrahigh vacuum (UHV) conditions.^5,6^

CO oxidation on metal surfaces is a critical heterogeneous catalytic process with significant implications in various industries. In particular, it plays a key role in converting CO into CO_2_ within automotive exhaust catalytic converters, thus contributing to emission control. In this respect, the behavior of ruthenium as a catalyst for this reaction has garnered significant interest due to its unusual characteristics compared to other transition metals like palladium, platinum, rhodium, and iridium. Although the catalytic performance of ruthenium for CO oxidation is notable when exposed to high gas pressures,^7−11^ under UHV conditions, CO oxidation cannot be thermally activated on this surface.^12^ However, experiments have shown that upon laser irradiation on the Ru(0001) surface with coadsorbed CO and O, both CO desorption and CO oxidation take place.^5,6,13^ It is observed that, still, CO desorption is much more likely than CO_2_ desorption, with a branching ratio of around 35 when the system is irradiated with an 800 nm laser pulse.

From a theoretical standpoint, examining these experiments requires conducting molecular dynamics simulations in an excited environment. First, the substrate excitation created by the laser is encapsulated by the two-temperature model (2TM), in which the excited system is described in terms of time-dependent electronic (*T*_e_(*t*)) and lattice (*T*_l_(*l*)) temperatures.^14^ Next, the adsorbate dynamics is determined by solving a set of coupled Langevin equations of motion, in which the effect of the electronic excitations/deexcitations is accounted for through the electronic friction force and related stochastic force that naturally depends on *T*_e_(*t*) .^15−24^ This can be performed at the level of density functional theory (DFT) by means of the so-called ab initio molecular dynamics with electronic friction and thermostats (*T*_e_, *T*_l_)-AIMDEF.^24−28^ In these latter simulations, the adiabatic forces on both adsorbates and surface atoms are calculated on the fly using the Hellmann–Feynman theorem.

A key limitation of (*T*_e_, *T*_l_)-AIMDEF is its substantial computational demand. In practice, this means that only a few hundred trajectories can be realistically computed for specific experimental conditions, leading to weak statistical power. Additionally, due to the required femtosecond time steps, the integration time is restricted to just a few picoseconds that may be insufficient to guarantee that the calculated desorption yields are converged, as discussed for CO photodesorption from Pd(111)^29−31^ and the recombinative photodesorption of different H_2_ isotopologues in Ru(0001) .^32^ In order to overcome these limitations, there has been an ever-growing interest in applying machine learning tools to generate potential energy surfaces (PESs) at the level of DFT accuracy from which adiabatic forces can be derived without having to invest so much computational resources. Among others, one can distinguish between kernel-based regression methods, such as the Gaussian approximation potential (GAP) model,^33^ and neural network-based methods. Regarding the latter, the majority of them belong to one of the two predominant representation choices. On the one hand, we have the atomistic neural networks first proposed by Behler and Parrinello,^34^ in which the total energy is decomposed into atomic contributions that are determined by the atomic local environment. The corresponding atomic descriptors must incorporate essential symmetries that systems must possess, such as translational, rotational, and permutational invariance. This can be achieved by following different strategies, such as, for instance, using sets of symmetric functions as done in the original work of Behler and Parrinello^34^ or more recent methods as the deep learning molecular dynamics (DPMD) model^35^ in which the local frame descriptors are used. On the other hand, a more contemporary approach to obtaining descriptors has emerged through the process of message passing.^36−40^ In this method, structure is represented as a graph where atoms serve as nodes and their distances (and sometimes enclosed angles) are typically incorporated in edge features. The descriptors are then optimized in a message passing way during the training of the neural network by minimizing deviations from the ground truth. Recently, both approaches had successes, not only with regards to metrics set by ab initio calculations but also with more complicated quantities of materials that necessitate the execution of molecular dynamics simulations.^31,32,41−44^

In the present work, we employ the embedded atom neural network (EANN) method,^45^ an atomistic neural network method based on embedded density descriptors, to construct an atomistic NNPES that allows us to study the laser-induced dynamics of CO and O coadsorbed on the Ru(0001) system. This problem was recently explored with (*T*_e_, *T*_l_)-AIMDEF.^25^ However, the aforementioned computational limitations of this methodology impeded the ability to extract definite conclusions regarding the role of thermal hot electrons in both the oxidation and desorption processes that can now be addressed with the NNPES. Thus, we start showing that the obtained NNPES can reproduce both static and dynamic metrics. Additionally, we go beyond the capabilities of ab initio molecular dynamics by calculating more than 30,000 trajectories, each with a duration of at least 30 ps, and therefore, we are finally able to obtain sufficient statistics for the oxidation reaction channel and perform a meaningful quantitative comparison to experiments. The semiquantitative agreement between the calculated desorption and oxidation probabilities and the 800 nm laser pulse experiments supports that the thermal (but nonequilibrated) hot electrons and phonons are responsible for both the photoinduced CO desorption and oxidation. Furthermore, the capability to simulate tens of picoseconds allows us to reveal the dynamic nature of the elusive CO precursor state to desorption that was identified in CO/Ru(0001) X-ray spectroscopy experiments^46,47^ but not in DFT calculations.^48,49^

## 2 Computational Methods

We start constructing the adiabatic NNPES for the Ru(0001) surface initially covered with 0.25 ML CO and 0.5 ML O (denoted as CO/2O/Ru(0001) in the following). Similar to other atomistic neural networks, the EANN method represents the total potential energy as a sum of the individual energies of the atoms in the system^45^
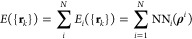
1where *N* is the total number of atoms and NN_*i*_ is the species-dependent atomic neural network of the *i*th atom. The local environment of each atom *i* is described in terms of the embedded density invariant at its position **ρ**^*i*^, which is given by the square of the linear combination of atomic Gaussian-type orbitals centered at its neighbors, i.e.
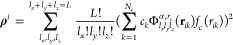
2and
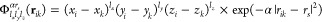
3

In these equations, **r**_*i*_ = (*x*_*i*_,*y*_*i*_,*z*_*i*_) and **r**_*k*_ = (*x*_*k*_,*y*_*k*_,*z*_*k*_) are the Cartesian coordinate vectors of the central atom *i* and neighbor atom *k*, respectively, where **r**_*ik*_ = **r**_*i*_ – **r**_*k*_ and *r*_*ik*_ = |**r**_*ik*_|; *r*_*s*_, α, and *L* = *l*_*x*_ + *l*_*y*_ + *l*_*z*_ are the center, width, and angular momentum of the Gaussian type orbitals, respectively; *f*_c_(*r*_*ik*_) is a cosine cutoff function that decays smoothly to zero as *r*_*ik*_ approaches the cutoff radius *r*_c_; *N*_c_ is the number of atoms within the cutoff radius. Finally, *c*_*k*_ are the element- and orbital-dependent weights that are adjusted during NN_*i*_ training. In this work, α was chosen to be 0.93 Å^–2^, while *r*_s_ was uniformly sampled in the interval [0, *r*_c_], where *r*_c_ = 6.5 Å. We utilized 15 Gaussian functions, considering angular momentum numbers from 0 to 3. Neural networks corresponding to each atom type have identical architecture and consist of two hidden layers of 60 neurons each.

**Figure 1:**
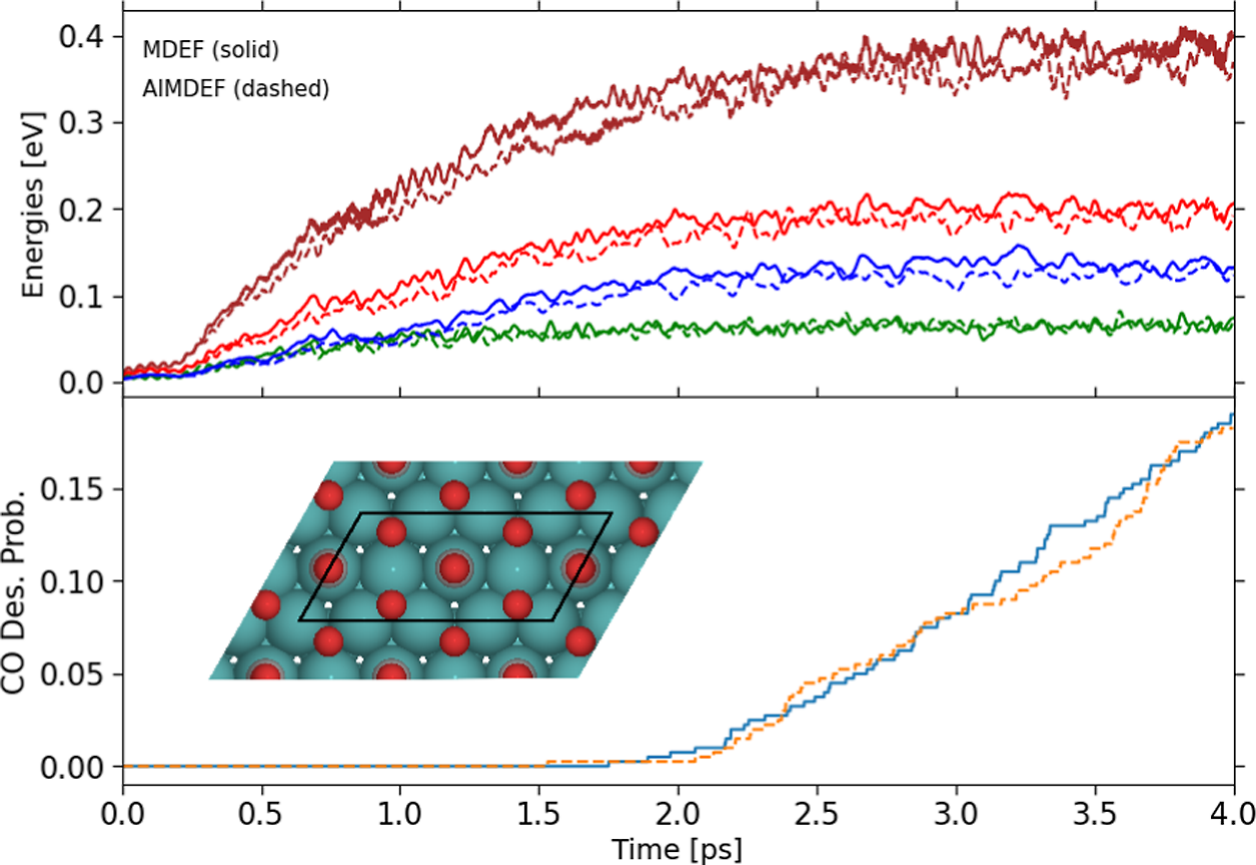
Top: Time evolution of the mean kinetic energy of CO adsorbed on CO/2O/Ru(0001) averaged over 200 trajectories as obtained in (*T*_e_, *T*_l_)-MDEF by using the NNPES to calculate the adiabatic forces (solid lines) and in (*T*_e_, *T*_l_)-AIMDEF^27^ (dashed lines). Results are shown for the mean total kinetic energy (brown), mean kinetic energy of the CO center of mass (red), and its contributions parallel (blue) and normal (green) to the surface. Bottom: Time evolution of the CO desorption probability for the same 200 trajectories as obtained in (*T*_e_, *T*_l_)-MDEF with the NNPES (blue) and in (*T*_e_, *T*_l_)-AIMDEF^27^ (orange). Inset: top view of the simulation cell delimited by the black parallelogram (O in red, C in gray, and Ru in blue). All results calculated for an 800 nm laser pulse with 110 fs fwhm and absorbed fluence *F* = 200 J/m^2^. The peak of the laser pulse is at 236 fs.

The training data set is constructed by taking advantage of 800,000 configurations that were obtained from (*T*_*e*_, *T*_l_)-AIMDEF simulations of the laser-induced dynamics of coadsorbed 0.25 ML CO and 0.5 ML O on the Ru(0001) surface.^25^ In those simulations, the system (adlayer and surface atoms) was initially thermalized at 100 K with the CO + 2O adlayer adopting the equilibrium honeycomb arrangement in which the atop CO is surrounded by six O atoms occupying the second nearest hcp and fcc sites^12,49^ (see inset in Figure 1). During each simulation, total energies and atomic forces were calculated with DFT and the van der Waals exchange–correlation functional proposed by Dion et al.^50^ The system was described using a periodic (4 × 2) surface unit cell (with 2 CO, 2 O on hcp, and 2 O on fcc sites) and five Ru layers. Each (*T*_*e*_, *T*_l_)-AIMDEF configuration contains information about the energy of the system, as well as the positions and forces of 48 atoms. While it might seem that there is an abundance of data points, it is essential to recognize that they may not adequately represent the entire configurational space of this complex system. This becomes apparent when categorizing trajectories based on desorption events. In a sample of 200 trajectories, 115 exhibit no desorption, 83 feature CO molecule desorption, and a mere 2 involve CO_2_ molecule desorption. Hence, to create a robust NNPES for molecular dynamics, a training set should be constructed with care in order to avoid the overrepresentation of a part of configurational space. For these reasons, we opt for training in an iterative fashion. First, 10,000 points are randomly chosen from the total data set, of which 90% was used for training and 10% for validation. Next, the accuracy of the converged NNPES is checked against the test data set that is formed by all the configurations not used in the corresponding training cycle. The training procedure is repeated by incorporating new points into the existing training set until reaching the required NNPES accuracy. To select which points from the test data set are added in each training cycle, we ranked them based on the following function
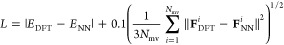
4where *E*_DFT_ (*E*_NN_) and **F**_DFT_^*i*^ (**F**_NN_^*i*^) are the total potential energy and force over atom *i* calculated with DFT (NNPES), respectively, with *N*_mv_ being the total number of moving atoms in the configuration (32 in our case). The number of points incorporated in each training cycle is equal to 5% of the training data set with the largest *L* values. At the end of the training process, the training set consisted of 248,147 points. Energy mean absolute error (MAE) and root-mean-square error (RMSE) on the remaining structures not used for training are 27.84 and 33.6 meV, respectively, with the corresponding values per atom being 0.58 and 0.70 meV. Atomic force MAE and RMSE per component are 128 and 138 meV/Å, respectively (see Supporting Information^51^ for more details on accuracy tests). It is very important to stress here that our trained neural network has only 22,503 parameters. This can be compared to some of the latest machine learning interaction potentials achieving state-of-the-art accuracy in reproducing extensive data sets of molecular properties, such as QM9,^52^ that often have millions or even tens of millions of trainable parameters.^53^ In doing that comparison, note that although QM9 contains information on a large variety of molecules, all this information refers to equilibrium properties. In contrast, a significant portion of our data set comprises configurations describing extreme out of equilibrium conditions, including two distinct processes, namely, CO desorption and CO oxidation.

Small errors in the training and test data sets, both consisting of static configurations, do not necessarily guarantee accurate results when using that NNPES to calculate the adiabatic forces in molecular dynamics simulations, among other things, because of the cumulative errors occurring during each trajectory propagation. Therefore, a further stringent accuracy test consists in verifying whether molecular dynamics simulations performed with the NNPES are able to reproduce the (*T*_e_, *T*_l_)-AIMDEF results for the laser-induced CO photodesorption and oxidation.

Similarly to (*T*_e_, *T*_l_)-AIMDEF, the laser-induced dynamics is modeled by combining the 2TM, which describes the excitation created by the laser, with Langevin equations of motion for adsorbates, in which the variance of the stochastic force directly depends on *T*_e_(*t*), and with Nosé–Hoover thermostat dynamics^54,55^ for surface atoms to ensure that they follow the lattice temperature *T*_l_(*t*) predicted by 2TM. The coupling of adsorbates to the hot electrons is realized within the local density friction approximation (LDFA),^56,57^ which requires knowledge of the surface electron density at the adsorbate position. The latter is calculated following the scheme proposed in refs (29 and 32), as detailed in the Supporting Information.^51^ A complete exposition of the above methodology (hereafter denoted (*T*_e_, *T*_l_)-MDEF whenever the adiabatic forces are calculated with the CO/2O/Ru(0001) NNPES) is not the purpose of this work and can be found elsewhere.^29^ In Figure 1, we compare the results of the (*T*_e_, *T*_l_)-MDEF dynamics based on the NNPES with those obtained previously in (*T*_e_, *T*_l_)-AIMDEF for the same set of 200 trajectories (initial conditions corresponding to the system thermalized at 100 K) and the same laser pulse. Specifically, the simulated laser pulse has a wavelength of 800 nm, a full width at half-maximum (fwhm) of 110 fs, and an absorbed fluence of 200 J/m^2^ as in experiments.^5^ Furthermore, as done in the (*T*_e_, *T*_l_)-AIMDEF simulations, a molecule (CO or CO_2_) is counted as desorbed if its center of mass height from the topmost Ru surface layer is ≥6.5 Å and its center of mass velocity along the surface normal is positive. In the top panel of Figure 1, we compare for the nondesorbing CO molecules the time evolution during the first 4 ps of the averaged total kinetic energy (brown), the average center of mass kinetic energy (red), and its contributions parallel (blue) and perpendicular (green) to the surface. The bottom panel shows the comparison of the time evolution of the CO desorption probability. The agreement achieved between the (*T*_e_, *T*_l_)-AIMDEF and the NNPES-based results for each of these quantities is remarkable. It demonstrates that the obtained NNPES allows us to perform dynamics at the ab initio level with a much lower computational cost.

## 3 Results and Discussion

**Figure 2:**
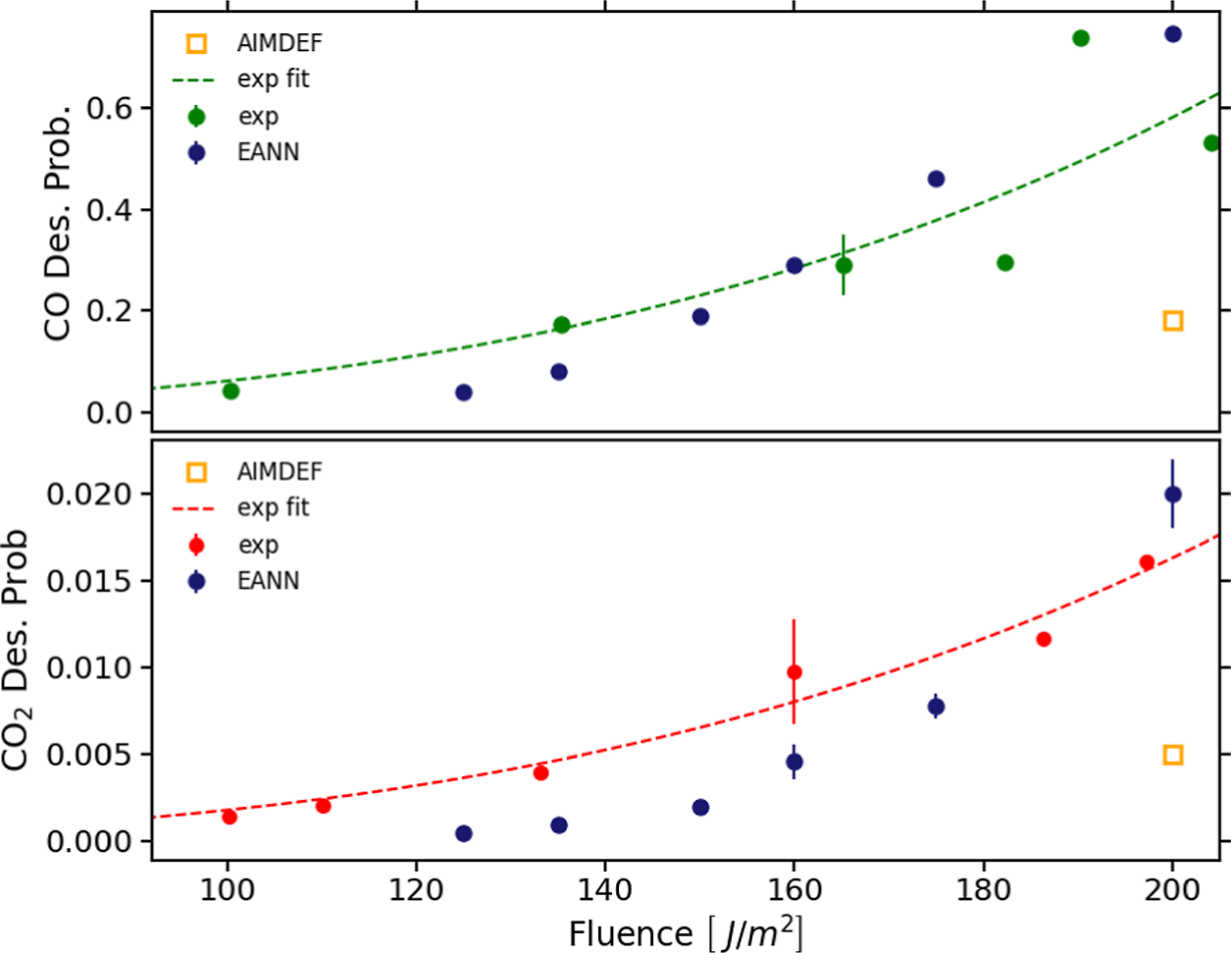
Fluence dependence of CO (top panel) and CO_2_ (bottom panel) desorption probabilities upon irradiating the CO/2O/Ru(0001) surface with an 800 nm laser pulse of fwhm 110 fs. Green (top panel) and red (bottom panel) circles are the experimental results shown in ref (58) for CO and CO_2_ desorption, respectively. Dashed lines in both panels are obtained by fitting the experimental data and should be used to guide the eye. Blue circles and error bars (the latter masked in many cases by the symbol size) are the results of our NNPES-based (*T*_e_, *T*_l_)-MDEF simulations. Open orange squares are the (*T*_e_, *T*_l_)-AIMDEF results for *F* = 200 J/m^2^.^25^

The (*T*_e_, *T*_l_)-AIMDEF simulations presented in ref (25) reproduced successfully some of the main features observed in experiments^5,6^ such as the existence of CO photodesorption and CO photooxidation (i.e., recombinative CO_2_ desorption), as well as the large branching ratio between desorption and oxidation that exceeds 1 order of magnitude. However, the large computational cost of the (*T*_e_, *T*_l_)-AIMDEF method imposed strong limitations regarding the quantitative comparison to experiments. More precisely, simulations were exclusively performed for one of the experimental absorbed laser fluences, namely *F* = 200 J/m^2^. From the calculated 200 trajectories, only two oxidation events were obtained. Hence, the calculated 0.5% probability for CO oxidation was not statistically meaningful. Moreover, the integration time was limited to 4 ps, but Figure 1 shows that the CO desorption probability curve is still increasing with time at this instant. Hence, it would be necessary to extend the integration time in the simulations until confirming that the desorption probabilities are well converged and quantitatively meaningful. The AIMDEF CO and CO_2_ desorption probabilities for *F* = 200 J/m^2^ are compared to experiments in Figure 2. As just discussed, the experimental CO to CO_2_ branching ratio is well reproduced, but the experimental probabilities are underestimated.

All the above limitations can be readily overcome in MDEF simulations by increasing the number of trajectories and integration time as necessary. Therefore, taking advantage of the probed quality and computational efficiency of the developed CO/2O/Ru(0001) NNPES, we investigate in this section the experimentally reported dependence of the CO desorption and CO oxidation probabilities on laser fluence by performing (*T*_e_, *T*_l_)-MDEF simulations for absorbed fluences in the range of *F* = 125–200 J/m^2^. The time-dependent electronic and lattice temperatures calculated for each *F*, as well as the parameters used in solving the 2TM equations, are detailed in the Supporting Information.^51^ In the simulations, the system is described with the periodic (4 × 2) surface cell shown in the inset of Figure 1 and a periodic slab that consists of the CO + 2O adlayer and five Ru layers. Each periodic Ru slab is separated by 300 Å along the surface normal. The number of trajectories (2500–7500) and integration time (30–50 ps) vary for each fluence to ensure accurate and converged results, allowing us a quantitative comparison to the experimental data (see a representative selection of time-resolved desorption probabilities in the Supporting Information^51^). Note that the large vacuum of the simulation cell assures a good description of the desorption process for the long integration times employed in our (*T*_e_, *T*_l_)-MDEF simulations. In these calculations, a molecule (CO or CO_2_) is counted as desorbed if its center of mass height from the topmost Ru surface layer is ≥10.5 Å,^59^ and its center of mass velocity along the surface normal is positive. In Figure 2, we show the results of our (*T*_e_, *T*_l_)-MDEF simulations for the CO (top panel) and CO_2_ (bottom panel) desorption probability as a function of the absorbed laser fluence, and we compare them to available experimental data.^58^ The calculated probabilities align remarkably well for both CO and CO_2_ desorption with the corresponding experimental data. First, as found in experiments, the desorption probability is significantly higher than the oxidation probability for all *F*. Second, both probabilities clearly exhibit the usual nonlinear dependence on fluence that characterizes photoinduced processes involving multiple electronic excitations.^3,4^ And, importantly, our statistically accurate and time-converged simulations are now able to reproduce not only qualitatively but also semiquantitatively the experimental data. In particular, it should be emphasized the excellent agreement achieved with experiment for the CO photooxidation process, especially considering the scarcity of oxidation events in the training set. Let us remark that the photodesorption probabilities are extremely sensitive to the minor details of the potential energy landscape. For this reason the agreement between theory and experiments found here can be qualified as very good, even if the theoretical points are not exactly matching the experimental ones. Altogether, our new (*T*_e_, *T*_l_)-MDEF results confirm that both CO desorption and CO oxidation are well understood in terms of the nonequilibrated but thermal hot electrons and phonons that are transiently created by the laser pulse.

**Figure 3:**
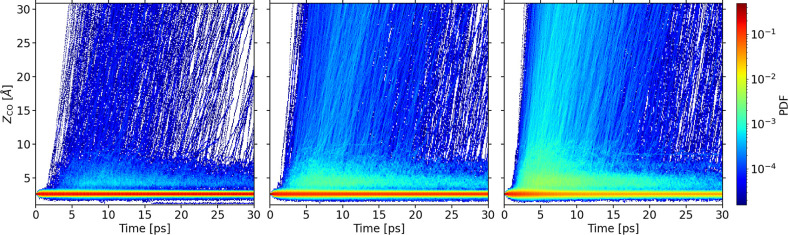
Normalized probability density of the CO center of mass height *Z*_CO_ as a function of time for three different absorbed laser fluences: 125 J/m^2^ (left), 160 J/m^2^ (middle), and 200 J/m^2^ (right). *Z*_CO_ is measured from the mean position of the Ru topmost layer. Bin area for all figures is 0.01 Å ps.

An interesting finding that could not be well observed during the first 4 ps that lasted the AIMDEF simulations is the eventual trapping of the desorbing CO in the physisorption region. At first sight, this result is surprising because, in contrast to other systems (CO/Au(111)^44,60^ and CO/Pd(111)^24,31^), the desorption path of a single CO along the surface normal is exclusively characterized by a deep chemisorption well of 1.57 eV, which is very similar to that on the 0.25 ML CO/Ru(0001) system.^48,49^Figure 3 shows the time evolution of the CO center of mass height *Z*_CO_ for three representative absorbed laser fluences that cover the whole *F*-range used in our calculations. The high probability density region at *Z*_CO_ ∼ 2.5 Å from the surface corresponds to the chemisorption region, i.e., to molecules that remain close to their initial adsorption well. We observe that the majority of desorption events take place during the first 10 ps after laser arrival. As already anticipated, another important observation corresponds to the high values of the probability density in the region *Z*_CO_ ∼ 4–5 Å from the surface. This feature corresponds to molecules dynamically trapped in the physisorption region. We have verified that some of these molecules remain trapped at the end of the simulations, whereas some others are finally desorbed. In other words, for this second group of molecules dynamic trapping constitutes a precursor state for desorption. Although the importance of this mechanism increases with *F*, it takes place for all the calculated fluences. In particular, the fraction of molecules that remain trapped within the region *Z*_CO_ = 3.5–5.5 Å at the end of the simulations varies between 0.17% at *F* = 125 J/m^2^ and 1% at *F* = 200 J/m^2^. Interestingly, the role played as a precursor to desorption by a physisorption state for CO/Ru(0001) was shown experimentally using X-ray emission and absorption spectroscopy.^46,47^ However, as aforementioned, no physisorption well nor dynamic trapping were found in previous CO/Ru(0001) DFT calculations of six dimensional PESs that considered simulation cells with a single CO molecule on the relaxed and nondistorted surface.^22,48^ No physisorption well was found either in static DFT + vdW-DF calculations performed in the same (4 × 2) simulation cell used here, in which the potential energy of the CO molecule at different distances from the surface on top of its chemisorption well was calculated.^49^ These previous theoretical results suggest that the trapping observed in our simulations is the result of the highly excited environment created by the laser pulse. In particular, all adsorbates become highly vibrationally excited, with CO tilting and both CO and O diffusing on the surface, while the Ru surface lattice becomes strongly distorted. The energy landscape seen by a desorbing CO under these extreme conditions changes drastically from that in equilibrium. Taking as an example one of these highly excited configurations of the system that are achieved several picoseconds after laser irradiation, and varying the center of mass height, (*X*, *Y*) position, and orientation of one of the CO molecules, we have verified the existence of a potential well in the physisorption region in which the dynamic trapping is taking place. In contrast, when performing a similar analysis, but for a slightly distorted surface configuration that corresponds to the system thermalized at 100 K, no physisorption well is realized. The mentioned two-dimensional cuts of the PES for the distorted surface and adlayer and for the surface at 100 K are shown in Figure S9 of the Supporting Information.^51^ This constitutes a proof of the dynamic character of the observed trapping and explains why the physisorption well does not appear in either static DFT calculations^49^ or in MDEF calculations using simulation cells with a single adsorbate in nondistorted surfaces.^22,48^

## 4 Conclusions

In summary, using the EANN method, we have constructed a NNPES trained on configurations extracted from previous (*T*_e_, *T*_l_)-AIMDEF simulations that is able to accurately describe the dynamics of coadsorbed O and CO on the Ru(0001) surface under laser irradiation. By describing the laser excitation by means of the 2TM and using the NNPES to perform molecular dynamics that accounts for both electronic and phononic excitations, we reproduce the results of short-time (*T*_e_, *T*_l_)-AIMDEF simulations for both the time-dependent kinetic energy of the adsorbates and the time-dependent CO desorption probability.

Since the molecular dynamics simulations performed on the NNPES are computationally much less expensive than the equivalent (*T*_e_, *T*_l_)-AIMDEF simulations, this allows us to calculate a much larger number of trajectories. In the present system, the latter is extremely important to gain valuable information on the CO_2_ recombinative desorption process. This is a very unlikely process, with probabilities below 2% for the typical absorbed laser fluences used in experiments. This means that, due to the computational cost, it is not possible to obtain statistical meaningful information using (*T*_e_, *T*_l_)-AIMDEF. Moreover, the NNPES-based simulations can be extended to longer times, which allows us to ensure the convergence of the results. The comparison of the results of the dynamics with the available experimental data is very satisfactory. First, the large branching ratio for CO desorption over CO_2_ desorption is reproduced. Second, we find very good agreement for the fluence dependence of the desorption yield of both CO and CO_2_. It must be emphasized that the employed approach has been finally able to obtain statistically meaningful results for the CO_2_ desorption mechanism at the level of DFT, which had not been possible up to now in previous dynamical studies.

Finally, by analyzing the movement of the molecules along the dynamics, we find the existence of dynamic trapping of CO that, in some cases, acts as a precursor state for CO desorption. Such a kind of precursor state was demonstrated to exist experimentally for CO photodesorption from Ru(0001), but it was not observed in previous molecular dynamics simulations performed in a six-dimensional PES. Here, we show that the energy landscape seen by a desorbing CO under equilibrium conditions and in the highly excited environment created by the laser pulse are drastically different, suggesting that the observed dynamic trapping is a consequence of the strong distortions and concomitant complex interactions created in the system.

All in all, our results demonstrate that the constructed NNPES based on the EANN method is a powerful tool to describe at the DFT level the dynamics of the CO/2O/Ru(0001) under strongly excited conditions. Its use has allowed us to perform statistically fully converged molecular dynamics simulations that finally confirm the validity of the description of the laser excitation in terms of non-equilibrated hot electrons and phonons, which nevertheless can be described by different temperatures, to understand the photoinduced desorption and oxidation of CO on metal surfaces.

## Supporting Information Available

(*T*_e_, *T*_l_)-AIMDEF simulations from which the training data set is obtained, comparison of energies and forces between NNPES and DFT, details on the (*T*_e_, *T*_l_)-MDEF simulations, parameters used in the 2TM, calculation of the friction coefficients, time-convergence tests, and observed trapping dynamics (PDF)
