# Supplementary material for: Understanding the Photoinduced Desorption and Oxidation of CO on Ru(0001) Using a Neural Network Potential Energy Surface
Source: JACS Au. 2024 May 10;4(5):1997–2004. doi: 10.1021/jacsau.4c00197 (PMC11134377; doi:10.1021/jacsau.4c00197)
Supplement: Supplementary file 1 — au4c00197_si_001.pdf [file au4c00197_si_001.pdf]

Supporting Information: Understanding the photoinduced desorption and oxidation of CO on Ru(0001) using a neural network potential energy surface

Ivan Žugec,[*,†] Auguste Tetenoire,[‡,‖] Alberto S. Muzas,[¶,†] Yaolong Zhang,[§] Bin Jiang,[§] Maite Alducin,[*,†,‡] and J. Iñaki Juaristi[*,†,‡,¶]

† *Centro de Física de Materiales CFM/MPC (CSIC-UPV/EHU), Paseo Manuel de Lardizabal 5, 20018 Donostia-San Sebastián, Spain*

‡ *Donostia International Physics Center (DIPC), Paseo Manuel de Lardizabal 4, 20018 Donostia-San Sebastián, Spain*

¶*Departamento de Polímeros y Materiales Avanzados: Física, Química y Tecnología, Facultad de Químicas (UPV/EHU), Apartado 1072, 20080 Donostia-San Sebastián, Spain*

§*Key Laboratory of Precision and Intelligent Chemistry, Department of Chemical Physics, University of Science and Technology of China, Hefei, Anhui 230026, China*

‖*Present address: Institut des Sciences Chimiques de Rennes - UMR CNRS 6226, F-35042 Rennes Cedex, France*

E-mail: zugec.ivan@gmail.com; maite.alducin@ehu.eus; josebainaki.juaristi@ehu.eus

S1 Brief description of the AIMDEF reference data

The (T_e,T_l)-AIMDEF simulations performed by Tetenoire et al.[S1] were carried out with VASP[S2,S3] (version 5.4) and the AIMDEF module[S4–S8] using a periodic supercell that consisted of a vector along the surface normal of 30.2225 Å that included about 19 Å of vacuum and the same (4×2) surface unit cell that we use here in (T_e,T_l)-MDEF. Within this supercell, the (0.25ML CO+0.5ML O)/Ru(0001) surface was described by five layers of Ru atoms and one (CO+2O) layer adsorbed on the topmost Ru surface layer. In the adlayer, each CO adsorbs atop a Ru atom, while the O atoms occupy the second nearest hcp and fcc sites forming a honeycomb arrangement around the CO.

During the AIMDEF simulations, the adiabatic forces were calculated with non spin-polarized DFT using the van der Waals exchange-correlation functional proposed by Dion et al.[S9] The equations of motion were integrated using a time step of 1 fs and the Beeman integrator implemented in both the AIMDEF module[S4] and the $(T_\mathrm{e}, T_\mathrm{l})$-MDEF dynamics code.[S10] On each integration step, the electronic ground state was determined by minimizing the system total energy up to a precision of 10^{-6} eV. Integration in the Brillouin zone was performed using a Γ-centered 3×6×1 Monkhorst-Pack grid of special **k** points[S11] and the Methfessel and Paxton scheme of first order with a broadening of 0.1 eV for occupations.[S12] An energy cut-off of 400 eV was used in the plane-wave expansion of the valence electrons, whereas the core electrons were treated with the projected augmented wave (PAW) method[S13] using the VASP implementation.[S14]

Using all the above parameters, 200 $(T_\mathrm{e}, T_\mathrm{l})$-AIMDEF trajectories with total simulation time of 4 ps each were run for the CO/2O/Ru(0001) surface. The latter was initially thermalized at 100 K and next irradiated with an 800 nm Gaussian pulse of 110 fs duration and an absorbed fluence $F = 200$ J/m^2. The $T_\mathrm{e}(t)$ and $T_\mathrm{l}(t)$ curves were calculated with the 2TM using the input parameters summarized in Table S2 of next section. The calculated curves for $F = 200$ J/m^2 are plotted in Figure S7.

S2 Accuracy of the EANN PES against the AIMDEF dataset

Figures S1 and S2 show the distribution of the potential energies and atomic force components (F_x, F_y, F_z) that form the final training data set. As explained in the main manuscript, the data correspond to configurations that were probed during the 200 (T_e, T_l)-AIMDEF trajectories with total simulation time of 4 ps each that were calculated in ref S1 for the CO/2O/Ru(0001) surface and an absorbed laser fluence of 200 J/m^2. The system can probe an ample configurational space that is characterized by a wide potential energy range of about 15.6 eV.

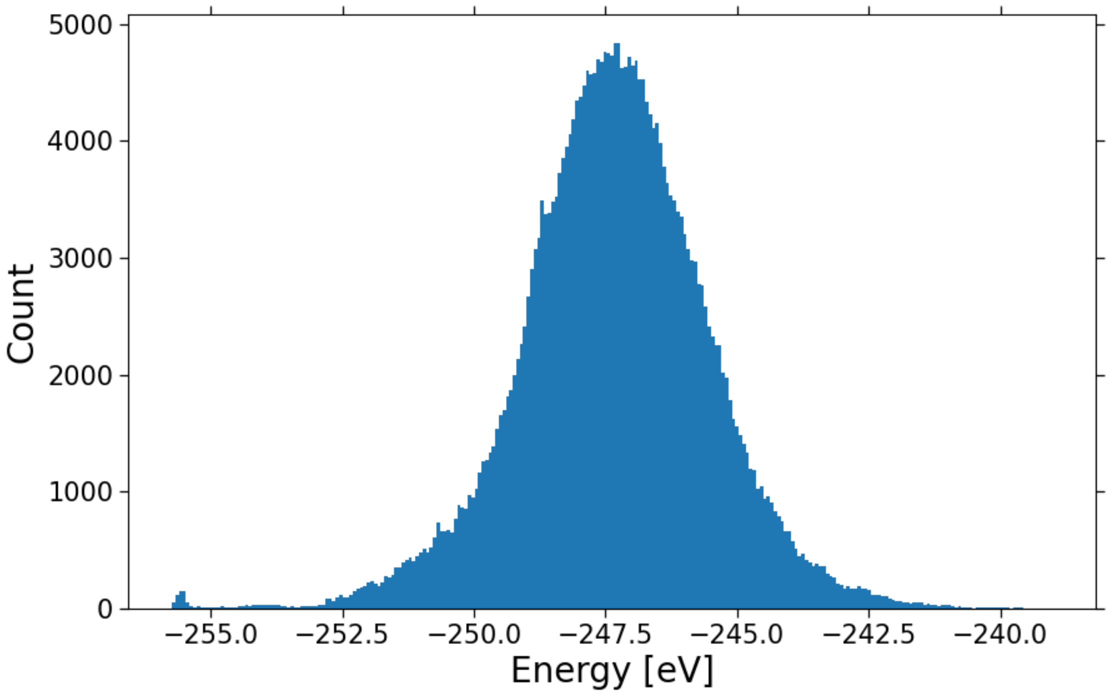

Figure S1: Distribution of the system potential energy probed during the 200 (T_e, T_l)-AIMDEF trajectories calculated in ref S1 for the CO/2O/Ru(0001) surface and F = 200 J/m^2.

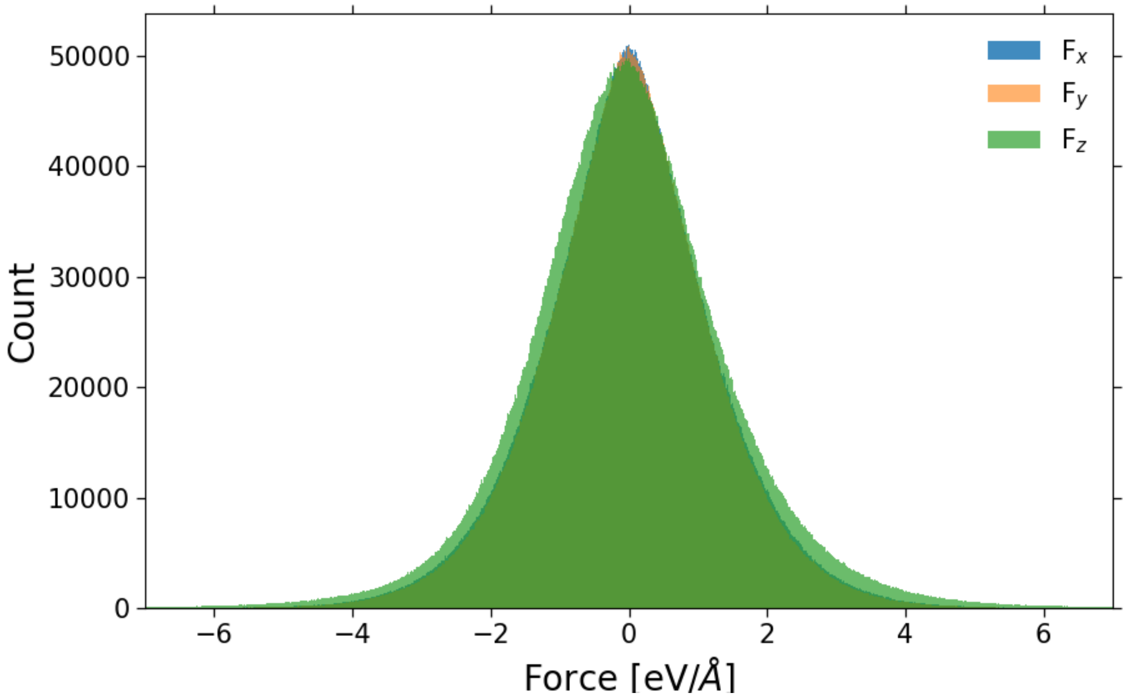

Figure S2: Same as Figure S1 but for each atomic force component.

Figures S3, S4, S5, and S6 show the comparison of the EANN-PES predicted energies and the three cartesian components of the atomic forces with the corresponding DFT values for the configurations of the test data set.

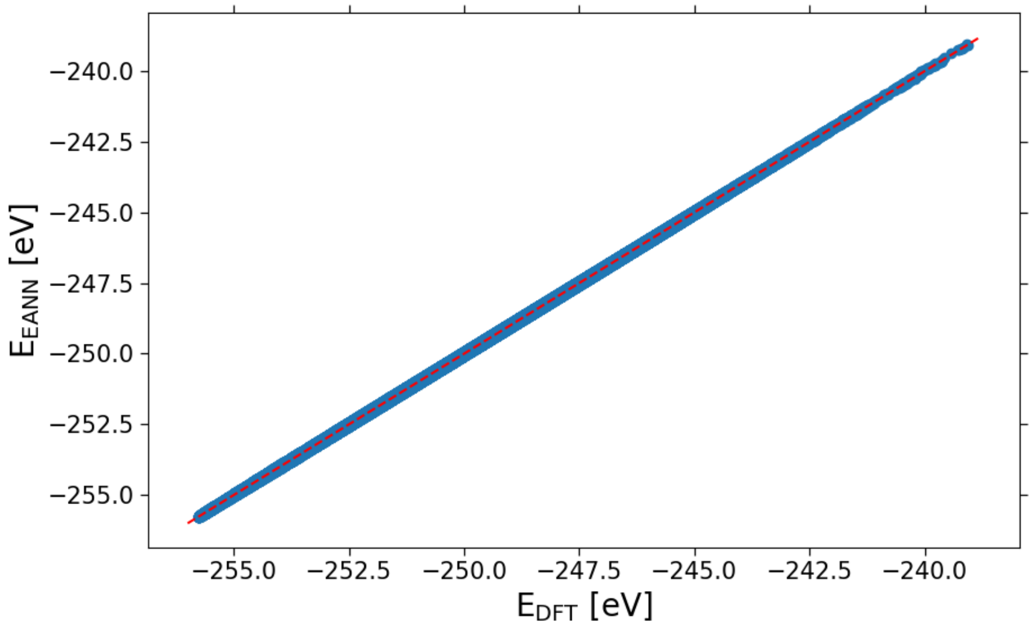

Figure S3: Comparison of the potential energies computed with the EANN-PES for the configurations in test data set and the corresponding DFT values.

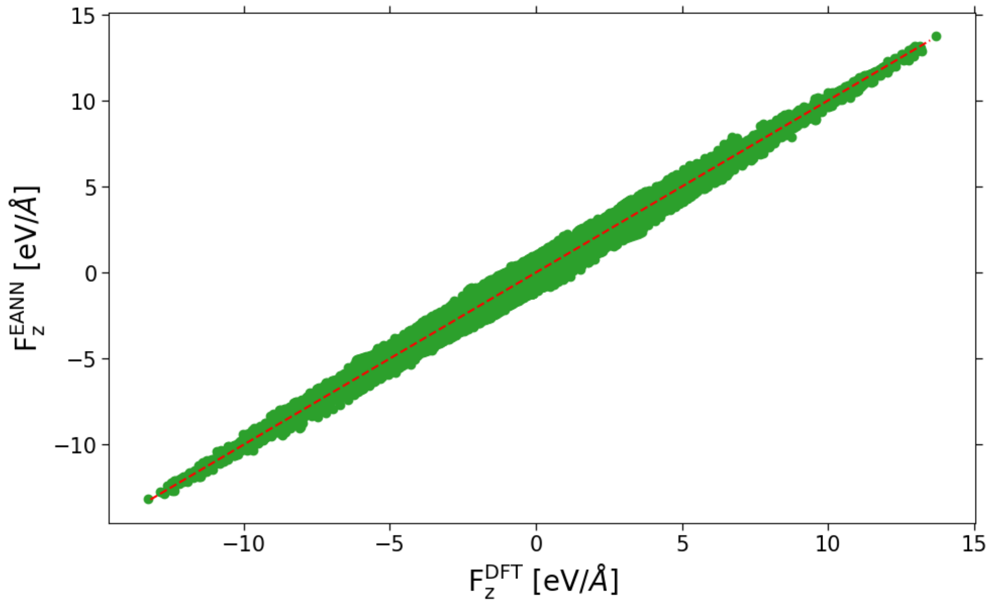

Figure S4: Comparison of the z component of the atomic forces computed with the EANN-PES for the configurations in test data set and the corresponding DFT values.

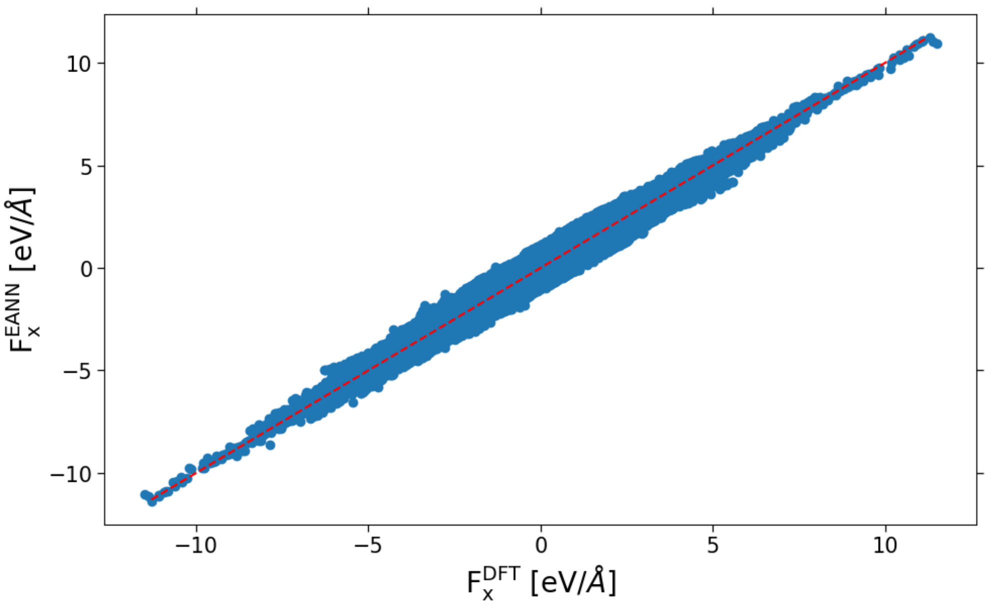

Figure S5: Comparison of the x component of the atomic forces computed with the EANN-PES for the configurations in test data set and the corresponding DFT values.

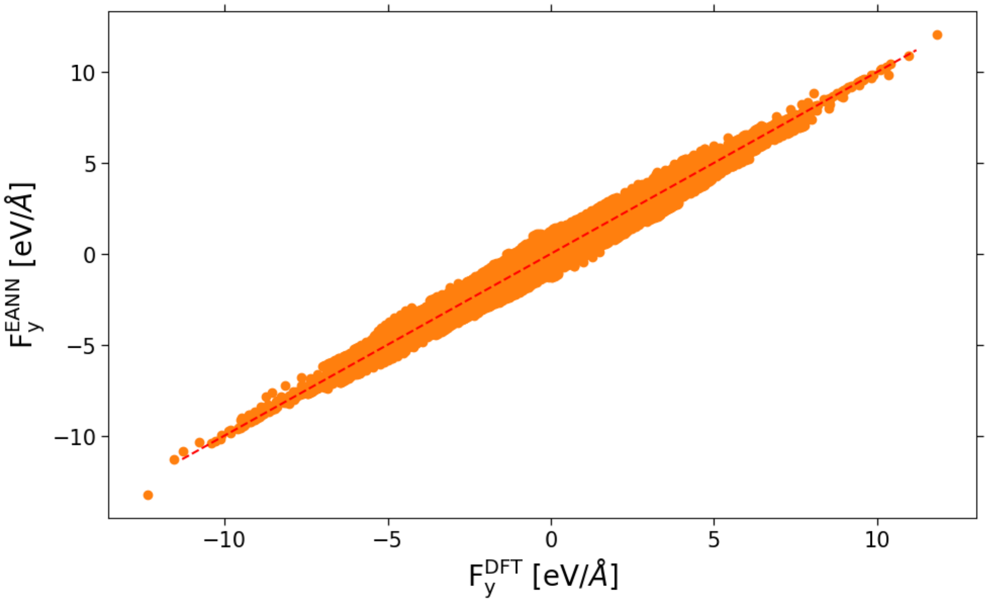

Figure S6: Comparison of the y component of the atomic forces computed with the EANN-PES for the configurations in test data set and the corresponding DFT values.

We have calculated using our EANN-PES the CO desorption energy and activation energy for CO oxidation in the equilibrium surface. These values compare rather well with the corresponding DFT data considering that no information on desorption or oxidation under equilibrium conditions was used for training the NNPES.

Table S1: CO desorption energy ($E_{des}(CO)$) and activation energy for CO oxidation ($E_a(CO_2)$) in the equlibrium surface (in eV) calculated with the EANN PES are compared to their corresponding values calculated with DFT+vdW-DF in S15.

	EANN PES	DFT+vdW-DF
$E_{des}(CO)$	1.49	1.57
$E_a(CO_2)$	1.16	1.19

S3 Laser-induced excitation: Two temperature model

All MDEF simulations were performed for the CO/2O/Ru(0001) surface being irradiated with an 800 nm Gaussian pulse of 110 fs duration (FWHM) and absorbed laser fluence in the range 125–200 J/m^2. Table S2 summarizes all the constants used to solve the following 2TM equations that describe the excitation created by the laser pulse in the Ru surface,

$$\begin{aligned} C_\mathrm{e}\frac{\partial T_\mathrm{e}}{\partial t} &= \frac{\partial}{\partial z}\kappa_\mathrm{e}\frac{\partial T_\mathrm{e}}{\partial z} - g\,(T_\mathrm{e} - T_\mathrm{l}) + S(z,t)\,, \\ C_\mathrm{l}\frac{\partial T_\mathrm{l}}{\partial t} &= g(T_\mathrm{e} - T_\mathrm{l})\,, \end{aligned} \tag{S1}$$

where C_e is the the electronic heat capacity that is assumed to depend linearly on T_e (i.e., $C_\mathrm{e} = \gamma_\mathrm{e} T_\mathrm{e}$), C_l is the phonon heat capacity described within the Debye model,[S16] κ_e is the electronic thermal conductivity described as $\kappa_\mathrm{e}(T_\mathrm{e}, T_\mathrm{l}) = \kappa_0 T_\mathrm{e}/T_\mathrm{l}$,[S16] g is the electron-phonon coupling parameter, z the perpendicular position relative to the surface, and $S(z,t)$ is the absorbed laser power per unit volume that for a Gaussian pulse of wavelength λ and absorbed fluence F is given by

$$S(z,t) = F\,\frac{e^{-z/\xi}}{\xi}\,\frac{e^{-(t-t_0)^2/(2\tau^2)}}{\sqrt{2\pi}\tau}\,. \tag{S2}$$

In this equation, $\tau = \mathrm{FWHM}/(2\sqrt{2\ln 2})$, t_0 is the instant at which the pulse intensity is maximum, and ξ is the light penetration depth of the metal surface for wavelength λ, which is calculated from the imaginary part of the reflective index k_λ as $\xi = \lambda/(4\pi k_\lambda)$. Recall that the above equations are implicitly assuming a constant absorbed laser fluence in the plane parallel to the metal surface and neglecting lattice thermal diffusion into the bulk. The diameter of the experimental laser beams and the time scale of interest justify each of those assumptions.[S17,S18]

The corresponding $T_\mathrm{e}(t)$ and $T_\mathrm{l}(t)$ curves are shown in Figure S7 for a representative selection of absorbed laser fluences.

Table S2: Constants and parameters used in the 2TM model for the Ru surface, taken from refs S19,S20.

Property	Value	Units
Initial temperature	100	K
Electron-phonon coupling parameter g	1.85×10^{18}	$W/(m^3K)$
Electronic heat capacity constant γ_e	400	$J/(m^3K^2)$
Thermal conductivity κ_0	117	W/(mK)
Debye temperature	600	K
Ru mass density	12370	kg/m^3
Optical penetration depth (800 nm)	15.6	nm

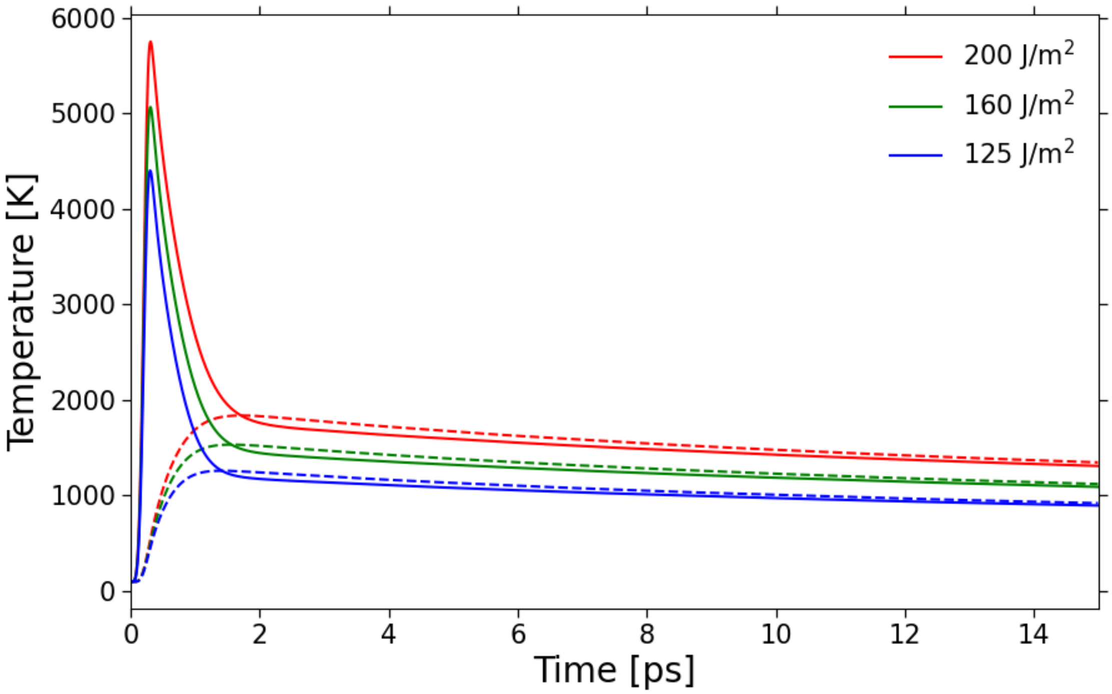

Figure S7: Electronic (solid lines) and lattice (dashed lines) temperatures calculated with the two temperature model for a Ru surface irradiated by an 800 nm Gaussian laser pulse, FWHM 110 fs, and various absorbed fluences F. The maximum of the laser pulse intensity is at $t = 236$ fs.

S4 Surface electron density

Within the Local Density Friction Approximation (LDFA)[S21,S22] used in our simulations, the electronic friction coefficient applied at each adsorbate depends on the value of the electronic density of the bare Ru(0001) surface at the position of the adsorbate, $\rho_0(\mathbf{R}_i)$. In the (T_e, T_l)-AIMDEF approach, $\rho_0(\mathbf{R}_i)$ is calculated using the Hirshfeld partitioning scheme[S23] to remove the adsorbate density contribution from the (DFT-calculated) self-consistent electron density of the complete system (adsorbates and Ru surface) that is calculated at each integration step (see refs S5,S6,S22 for details). Conversely, in (T_e, T_l)-MDEF the electron density of the bare Ru(0001) surface at the position of each adsorbate is modeled as a sum of the electronic densities contributed by individual Ru atoms at this position. The contribution of each Ru atom to the density is described by two decaying exponential functions. As a result, the expression for $\rho_0(\mathbf{R}_i)$ is the following:

$$\rho_0\left(\mathbf{R}_i\right) = \sum_{\mathrm{Ru}} \left(a \exp\left(-b\left|\mathbf{R}_i - \mathbf{r}_{Ru}\right|\right) + c \exp\left(-d\left|\mathbf{R}_i - \mathbf{r}_{Ru}\right|\right)\right) , \tag{S3}$$

where $\mathbf{r}_{Ru}$ is the position of the Ru atom. After fitting this function to the electronic density data extracted from the (T_e, T_l)-AIMDEF simulations, the values of the parameters are: $a = 4.178222$ a.u., $b = 4.022414$ Å^{-1} , $c = 0.061987$ a.u., and $d = 1.876455$ Å^{-1} (a.u. stands for atomic units).

S5 Statistics of the (T_e,T_l)-MDEF simulations

The specific number of trajectories run for each absorbed laser fluence as well as the total simulation time used in each case are given in Table S3. It is worth to remark that a single (T_e, T_l)-AIMDEF trajectory with a simulation time of 4 ps and a time step of 1 fs (i.e., 4000 integration steps) took between 5-7 days on 24 cores (Intel(R) Xeon(R) Platinum 8168 CPU @ 2.70GHz), whereas a single (T_e, T_l)-MDEF trajectory with a simulation time of 30

ps and a time step of 0.5 fs (i.e., 60 000 integration steps) takes between 2 to 3 minutes on 36 cores (mixed cluster with Intel(R) Xeon(R) Platinum and Intel(R) Xeon(R) Gold, CPUs @ 2.70GHz-3.00GHz).

Table S3: Details of the (T_e, T_l)-MDEF simulations carried out for each absorbed laser fluence: number of trajectories N_{traj} and simulation time t_{end}.

Fluence (J/m^2)	125	135	150	160	175	200
N_{traj}	7500	5000	7500	2500	7400	2500
$t_{end}(ps)$	30	30	30	30	30	50

S6 Time-convergence of the Desorption Probability

As defined in the Results and Discussion section of the main text, in the (T_e,T_l)-MDEF simulations presented there, a molecule is considered to be desorbed if its center of mass height from the topmost Ru surface layer is ≥ 10.5 Å and its center of mass velocity along the surface normal is positive. Following this definition, the time evolution of the desorption probability is calculated at each integration step t as

$$P_{des}(t) = \frac{1}{N_{traj}\, N_{CO}} \sum_{i=1}^{N_{traj}} N_{des,i}(t) \tag{S4}$$

where N_{traj} and N_{CO} are the total number of trajectories for a given fluence and total number of CO molecules present in the unit cell, respectively. $N_{des,i}(t)$ is the number of desorbed molecules at the time-step t for a given trajectory i.

Figure S8 shows the time evolution of the CO and CO_2 desorption probabilities for the representative absorbed fluences, 125 J/m^2, 160 J/m^2, and 200 J/m^2. The values of the CO and CO_2 desorption probabilities that are compared to experimental data correspond to the values obtained at the end of simulation time for each fluence, $P_{des}(t = t_{end})$. As shown in this figure, all the $P_{des}(t)$ curves are well saturated at the end of the simulation time, demonstrating that the desorption probabilities reported in the Results and Discussion

section of the main text are fully converged respect to time.

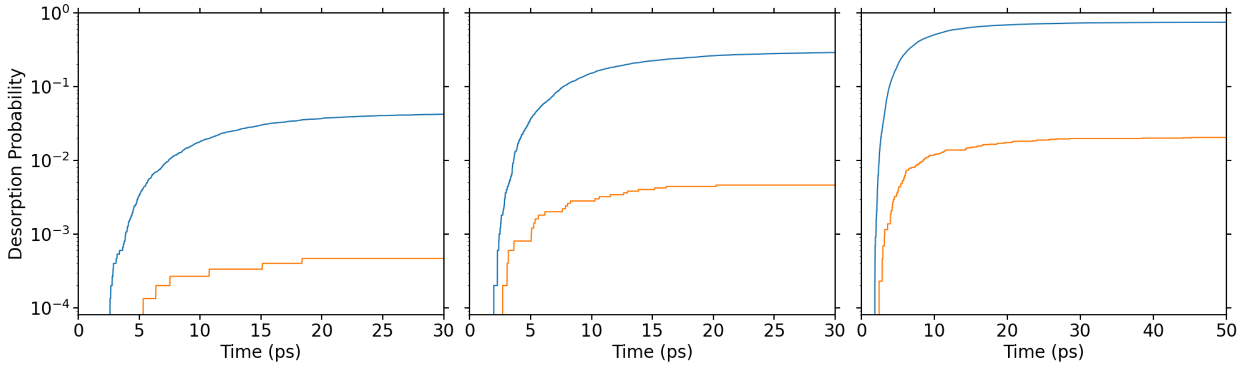

Figure S8: Time dependent CO desorption probability (blue) and CO_2 desorption probability (orange) for three representative absorbed laser fluences that cover the whole F-range used in our calculations: 125 J/m^2 (left), 160 J/m^2 (middle), and 200 J/m^2 (right).

The error bars of the calculated desorption probabilities shown in Figure 2 are calculated as the standard error (SE) of the corresponding probability at the end of the simulation, i.e.,

$$\mathrm{SE}\left[P_{\mathrm{des}}(t_{\mathrm{end}})\right] = \sqrt{\frac{\mathrm{Var}\left[P_{\mathrm{des}}(t_{\mathrm{end}})\right]}{N_{\mathrm{traj}}}}\,, \tag{S5}$$

where the variance is obtained as

$$\mathrm{Var}\left[P_{\mathrm{des}}(t_{\mathrm{end}})\right] = \frac{1}{N_{\mathrm{traj}}-1}\sum_{i=1}^{N_{\mathrm{traj}}}\left(\frac{N_{\mathrm{des},i}(t_{\mathrm{end}})}{N_{\mathrm{CO}}} - P_{\mathrm{des}}(t_{\mathrm{end}})\right)^2 \tag{S6}$$

S7 Dynamic trapping

An interesting finding visible in Figure 3 of the main text is the dynamic trapping of CO molecules. In order to understand this behaviour, analysis was conducted by displacing the center of mass of one CO molecule in the $x-y$ plane over a range of CO center-of-mass heights from the ruthenium surface, $Z_{\mathrm{cm}} = 3 - 6.5$ Å. For each CO height and position over the surface plane, the polar angle of the molecular axis was varied within the range [0 - π]. Figure S9 illustrates an example of an energy landscape calculated by the NNPES for the thermalized, slightly perturbed surface at 100 K, and for a distorted surface taken from one

of the MDEF trajectories exhibiting CO dynamics trapping. The dark blue area observed in the top-left panel at $Z_{\mathrm{cm}} = 3.1$ Å corresponds to the chemisorption well of the CO that is being displaced, while the orange-red areas also observed at $Z_{\mathrm{cm}} = 4.5$ Å indicate CO-CO repulsion. For the distorted surface, the physisorption well can be clearly observed in the right-middle panel as the dark-blue area.

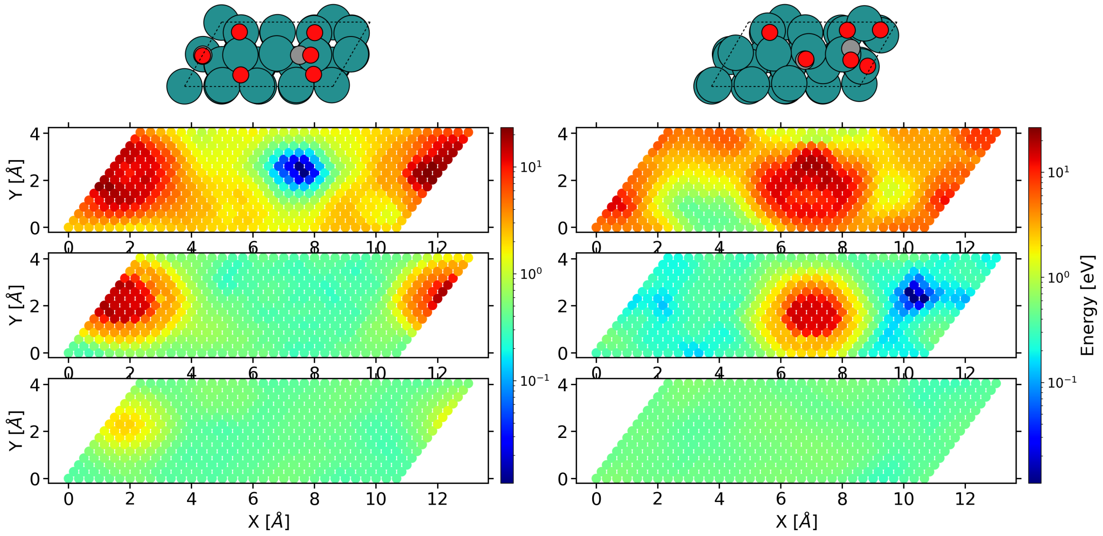

Figure S9: 2D cuts of the potential energy surface calculated by the NNPES for the thermalized surface at 100 K (left), and for a distorted surface taken from one of the MDEF trajectories exhibiting CO dynamics trapping (right). The cuts were calculated at distances of the CO center of mass from the ruthenium surface, $Z_{\mathrm{cm}} = 3.1$ Å (top panels), $Z_{\mathrm{cm}} = 4.5$ Å (middle panels), and $Z_{\mathrm{cm}} = 6$ Å (bottom panels), respectively. In all cases, the polar angle of the molecular axis for displaced CO molecule is $\pi/4$. Top views of the surface unit cell for the thermalized and distorted surfaces are shown atop the left and right panels, respectively. In both cases, the CO that is being displaced to create the 2D plots is the rightmost one.

Acknowledgement

The authors acknowledge financial support by the Spanish MCIN/AEI/ 10.13039/501100011033/ and FEDER "Una manera de hacer Europa" [Grant No. PID2022-140163NB-I00], Gobierno Vasco-UPV/EHU [Project No. IT1569-22], and the Basque Government Education Department IKUR program, also cofunded by the European NextGener-

ationEU action through the Spanish PRTR. This research was conducted in the scope of the Transnational Common Laboratory (LTC) "QuantumChemPhys – Theoretical Chemistry and Physics at the Quantum Scale". Computational resources were provided by the DIPC computing center.

References

(S1) Tetenoire, A.; Ehlert, C.; Juaristi, J. I.; Saalfrank, P.; Alducin, M. Why Ultrafast Photoinduced CO Desorption Dominates over Oxidation on Ru(0001). *J. Phys. Chem. Lett.* **2022**, *13*, 8516–8521.

(S2) Kresse, G.; Furthmüller, J. Efficiency of Ab-Initio Total Energy Calculations For Metals and Semiconductors Using a Plane-Wave Basis Set. *Comput. Mater. Sci.* **1996**, *6*, 15 – 50.

(S3) Kresse, G.; Furthmüller, J. Efficient Iterative Schemes For Ab Initio Total-Energy Calculations Using a Plane-Wave Basis Set. *Phys. Rev. B.* **1996**, *54*, 11169–11186.

(S4) Blanco-Rey, M.; Juaristi, J. I.; Díez Muiño, R.; Busnengo, H. F.; Kroes, G. J.; Alducin, M. Electronic Friction Dominates Hydrogen Hot-Atom Relaxation on Pd(100). *Phys. Rev. Lett.* **2014**, *112*, 103203.

(S5) Novko, D.; Blanco-Rey, M.; Juaristi, J. I.; Alducin, M. *Ab Initio* Molecular Dynamics with Simultaneous Electron and Phonon Excitations: Application to the Relaxation of Hot Atoms and Molecules on Metal Surfaces. *Phys. Rev. B.* **2015**, *92*, 201411.

(S6) Novko, D.; Blanco-Rey, M.; Alducin, M.; Juaristi, J. I. Surface Electron Density Models for Accurate *Ab Initio* Molecular Dynamics with Electronic Friction. *Phys. Rev. B.* **2016**, *93*, 245435.

(S7) Novko, D.; Lončarić, I.; Blanco-Rey, M.; Juaristi, J. I.; Alducin, M. Energy Loss

and Surface Temperature Effects in Ab Initio Molecular Dynamics Simulations: N Adsorption on Ag(111) as a Case Study. *Phys. Rev. B* **2017**, *96*, 085437.

(S8) Juaristi, J. I.; Alducin, M.; Saalfrank, P. Femtosecond Laser Induced Desorption of H_2, D_2, and HD from Ru(0001): Dynamical Promotion and Suppression Studied with Ab Initio Molecular Dynamics with Electronic Friction. *Phys. Rev. B* **2017**, *95*, 125439.

(S9) Dion, M.; Rydberg, H.; Schröder, E.; Langreth, D. C.; Lundqvist, B. I. Van Der Waals Density Functional for General Geometries. *Phys. Rev. Lett.* **2004**, *92*, 246401.

(S10) Serrano-Jiménez, A.; Muzas, A. P. S.; Zhang, Y.; Ovčar, J.; Jiang, B.; Lončarić, I.; Juaristi, J. I.; Alducin, M. Photoinduced Desorption Dynamics of CO from Pd(111): A Neural Network Approach. *J. Chem. Theory Comput.* **2021**, *17*, 4648–4659.

(S11) Monkhorst, H. J.; Pack, J. D. Special Points for Brillouin-Zone Integrations. *Phys. Rev. B* **1976**, *13*, 5188–5192.

(S12) Methfessel, M.; Paxton, A. T. High-Precision Sampling for Brillouin-Zone Integration in Metals. *Phys. Rev. B* **1989**, *40*, 3616–3621.

(S13) Blöchl, P. E. Projector Augmented-Wave Method. *Phys. Rev. B* **1994**, *50*, 17953–17979.

(S14) Kresse, G.; Joubert, D. From Ultrasoft Pseudopotentials to the Projector Augmented-wave Method. *Phys. Rev. B* **1999**, *59*, 1758–1775.

(S15) Tetenoire, A.; Juaristi, J. I.; Alducin, M. Insights Into the Coadsorption and Reactivity of O and CO on Ru (0001) and Their Coverage Dependence. *J. Phys. Chem. C* **2021**, *125*, 12614–12627.

(S16) Ashcroft, N. W.; Mermin, N. D. *Solid State Physics*; Holt-Saunders, 1988.

(S17) Frischkorn, C.; Wolf, M. Femtochemistry at Metal Surfaces: Nonadiabatic Reaction Dynamics. *Chem. Rev.* **2006**, *106*, 4207–4233.

(S18) Saalfrank, P. Quantum Dynamical Approach to Ultrafast Molecular Desorption from Surfaces. *Chem. Rev.* **2006**, *106*, 4116–4159, PMID: 17031982.

(S19) Bonn, M.; Denzler, D. N.; Funk, S.; Wolf, M.; Wellershoff, S.-S.; Hohlfeld, J. Ultrafast Electron Dynamics at Metal Surfaces: Competition Between Electron-Phonon Coupling and Hot-Electron Transport. *Phys. Rev. B* **2000**, *61*, 1101–1105.

(S20) Vazhappilly, T.; Klamroth, T.; Saalfrank, P.; Hernandez, R. Femtosecond-Laser Desorption of $H_2(D_2)$ from Ru(0001): Quantum and Classical Approaches. *J. Phys. Chem. C* **2009**, *113*, 7790–7801.

(S21) Juaristi, J.; Alducin, M.; Díez Muiño, R.; Busnengo, H. F.; Salin, A. Role of Electron-Hole Pair Excitations in the Dissociative Adsorption of Diatomic Molecules on Metal Surfaces. *Phys. Rev. Lett.* **2008**, *100*, 116102.

(S22) Alducin, M.; Díez Muiño, R.; Juaristi, J. I. Non-Adiabatic Effects in Elementary Reaction Processes at Metal Surfaces. *Prog. Surf. Sci.* **2017**, *92*, 317 – 340.

(S23) Hirshfeld, F. XVII. Spatial Partitioning of Charge Density. *Israel Journal of Chemistry* **1977**, *16*, 198–201.
